# Determination of skeletal muscle mass by aspartate aminotransferase / alanine aminotransferase ratio, insulin and FSH in Chinese women with sarcopenia

**DOI:** 10.1186/s12877-022-03491-9

**Published:** 2022-11-22

**Authors:** Mengting Yin, He Zhang, Qianhui Liu, Fei Ding, Lisha Hou, Yiping Deng, Tao Cui, Yixian Han, Yijun Chen, Chen Huang, Jirong Yue, Yong He

**Affiliations:** 1 Department of Laboratory Medicine, West China Hospital, Sichuan University, Chengdu, Sichuan Province China; 2 Department of Geriatrics and National Clinical Research Center for Geriatrics, West China Hospital, Sichuan University, Chengdu, Sichuan Province China; 3 Department of Gynecology and Obstetrics, West China Second University Hospital, Sichuan University, Chengdu, Sichuan Province China; 4 Key Laboratory of Birth Defects and Related Diseases of Women and Children (Sichuan University), Ministry of Education, Chengdu, Sichuan Province China; 5 Department of Laboratory Medicine, West China Hospital of Sichuan University, 37 Guoxue Xiang, 610041 Chengdu, Sichuan China; 6 Department of Geriatrics, West China Hospital, National Clinical Research Center for Geriatrics, Sichuan University, 610041 Chengdu, Sichuan China

**Keywords:** Sarcopenia, Muscle mass, FSH, Insulin, AST/ALT ratio

## Abstract

**Background:**

Sarcopenia is an age-related skeletal muscle disorder that involves a loss of muscle mass or strength and physiological function. Skeletal muscle deteriorates in both quantity and quality. The endocrine system is an important regulator of muscle metabolism. Therefore, we aimed to explore the relationship between biochemical markers and muscle mass in sarcopenia.

**Methods:**

We used the AWGS 2014 as the diagnostic criteria for sarcopenia, considering both the loss in muscle mass, muscle strength and physical performance. A total of 2837 elderly female participants over 50 years of age from the West China Health and Aging Trend (WCHAT) study were included. Insulin, glucose, 25(OH)VD, procalcitonin, alanine aminotransferase, aspartate aminotransferase, total protein, prealbumin, albumin, thyroid-stimulating hormone, free triiodothyronine, free tetraiodothyronine, triglycerides, cholesterol, high-density lipoprotein, very low-density lipoprotein, cortisol, and follicle-stimulating hormone were measured. Based on the findings of univariate analysis, multivariate regression and receiver operating characteristic (ROC) curves were established.

**Results:**

Participants with sarcopenia had significantly lower free triiodothyronine, insulin, total protein, albumin, prealbumin, albumin/prealbumin ratio (A/G), alanine aminotransferase, triglycerides, and very low-density lipoprotein concentrations (*P* < 0.05). Compared with those without sarcopenia, those with sarcopenia had significantly higher free tetraiodothyronine, cortisol, follicle-stimulating hormone (FSH), aspartate aminotransferase/alanine aminotransferase ratio (AST/ALT), and high-density lipoprotein concentrations (*P* < 0.05). Insulin (OR = 0.854), FSH (OR = 1.016), and the AST/ALT ratio (OR = 1.819) were independent risk factors for low muscle mass (*P* < 0.001). The AUC of insulin was the highest, followed by the AST/ALT ratio and FSH (0.691, 0.671, and 0.634, respectively), and the AUC of the mixture of the above three reached 0.736.

**Conclusion:**

In this cross-sectional study of elderly Chinese females aged over 50 years from the WCHAT, FSH, insulin, and AST/ALT ratio were associated with sarcopenia and risk factors for low muscle mass.

## Introduction

Skeletal muscle is one of the largest organs in the human body, and the weight of skeletal muscle is approximately 30–40% of the human body [1]. Skeletal muscle mass gradually decreases with aging as early as 50 years of age [2]. Sarcopenia is an age-related skeletal muscle disorder involving the loss of muscle mass or strength and physiological function [3]. Currently, many organizations have proposed diagnostic criteria based on muscle mass and muscle function with various cutoffs and different measurement tools [4–7]. Although a range of body imaging techniques have been widely used in the measurement of appendicular skeletal muscle mass, such as magnetic resonance imaging, dual-energy X-ray absorptiometry, bioelectric impedance analysis, and computed tomography, there is a limitation that the diagnostic procedures for body composition estimation and muscle function need to be inexpensive, convenient and standardized. Meanwhile, sarcopenia is a multifactorial disease, and the pathophysiology not only includes muscle loss but is also associated with endocrine and metabolic abnormalities [8–10]. A series of biomarkers in blood samples have been proven to play a role in the development of sarcopenia [11]. It has been reported that follicle-stimulating hormone (FSH) has an impact on bone loss both in human and animal studies [12–14]. Park et al. analyzed the skeletal mass across the stages of the menopause transition in women and found that appendicular lean mass of menopausal women was inversely related to FSH levels but not estradiol [12]. However, Wu et al. elucidated that FSH levels were not associated with bone mass or body composition changes in either older men or women[15]. Therefore, we focused on the correlation of blood biochemical markers and hormones with skeletal muscle mass in the female sarcopenia cohort.

## Methods

### Study participants

Data were selected from the baseline of the West China Health and Aging Trend (WCHAT) study, which was initiated from July to December 2018 and included 7536 people aged 50 or older in Sichuan, Yunnan, Guizhou, and Xinjiang Provinces. Multistage cluster sampling was applied, and the total response rate was 50.2% [16, 17]. Patients with cognitive impairment, a recent history of malignancy, missing data, and male participants were excluded, resulting in a total of 2837 elderly patients, 497 of whom were diagnosed with sarcopenia. All participants were willing to take part in this study, and informed consent was signed. The study was conducted in accordance with the Declaration of Helsinki and was approved by the Ethics Committee of Sichuan University and is supported by Grant No. 2020YFC2005600 from the National Key R&D Program of China.

### Sarcopenia assessment

Muscle mass was measured by bioimpedance analysis using an Inbody 770 (BioSpace, Seoul, Korea), which is well accepted and validated in the Chinese population [18–20]. The subject stands with the soles in contact with the foot electrodes and grabs the hand electrodes. The sequence of the measurements, controlled by a microprocessor, reports on the screen and paper. No precaution was taken to standardize the subject’s posture before BIA, as suggested by the manufacturer. RI values were calculated at all frequencies. Data output, as calculated by using the manufacturer’s algorithm, included fat mass and skeletal muscle mass of the total body, arms, and legs. Muscle strength was assessed with the dominant hand using a dynamometer (EH101; Camry, Zhongshan, China). The participants were asked to exert maximum effort in a standing position, two readings were taken from each side, and the maximum value from the dominant hand. The Asia Working Group for Sarcopenia (AWGS) 2014 was used to identify sarcopenia [21]. Appendicular skeletal muscle mass index (ASMI, ASM/height^2^) (male: <7.0 kg/m^2^, female: <5.7 kg/m^2^) for bioimpedance analysis is considered low muscle mass. The AWGS also suggests that handgrip strength of < 26 kg and < 18 kg for men and women is defined as low muscle strength. A 4 m walking test < 0.8 m/s is considered a low gait speed. Patients were diagnosed with sarcopenia when low muscle strength and poor muscle function were confirmed. Meanwhile, if low physical performance is also accompanied, sarcopenia is considered severe.

### Specimen collection

Fasting venous blood samples were drawn in the morning, with the participants in a sitting position. Fasting insulin, glucose, 25(OH)VD, procalcitonin, alanine aminotransferase, aspartate aminotransferase, total protein, prealbumin, albumin, thyroid-stimulating hormone, free triiodothyronine, free tetraiodothyronine, triglycerides, cholesterol, high-density lipoprotein, very low-density lipoprotein, plasma total cortisol, and follicle-stimulating hormone were measured.

### Statistical analysis

Measurement data of continuous variables are presented as the mean ± SD, and categorical variables are expressed as frequencies with percentages. The Kolmogorov–Smirnov test was used to confirm the normal distribution. The difference between groups was tested by *t* test for normally distributed data and the Mann–Whitney U test for abnormally distributed variables. The chi-square test was used for categorical variables. MedCalc v19.0.7 was used to evaluate the receiver operating characteristic (ROC) curve. Multivariate regression was used to identify risk factors for the low muscle mass. Spearman correlation analysis was also established to identify the relationship between skeletal muscle mass and biochemical markers. Variables with statistical differences between groups were selected for the multivariate regression and spearman correlation. All statistical tests were two-sided, and a *P* value < 0.05 was considered statistically significant. All other statistical analyses were done using SPSS 19.0.

## Results

### Characteristics of the study cohorts

Among the 2837 female participants, 497 had sarcopenia, with an age of 67.2 ± 9.0. Table 1 summarizes the blood biochemical markers and hormones of the elderly participants stratified by sarcopenia. Patients with sarcopenia had a worse nutritional status than healthy individuals. Compared with those without sarcopenia, those with sarcopenia had lower TP (71.6 ± 5.3 vs. 72.3 ± 6.3), ALB (43.3 ± 3.3 vs. 44.5 ± 2.9), and PA (257.31 ± 126.01 vs. 278.38 ± 148.23), (*P* < 0.01). Sarcopenia participants had lower values of TG (1.74 ± 1.65 vs. 1.92 ± 1.76) and VLDL (0.79 ± 0.75 vs. 0.87 ± 0.80) but higher values of HDL (1.39 ± 0.35 vs. 1.29 ± 0.29) than healthy participants in terms of lipid metabolism (*P* < 0.05). In addition, in the hormone state, the sarcopenia group had lower FT3 (4.30 ± 0.73 vs. 4.52 ± 1.43) but higher FT4 (18.06 ± 2.98 vs. 17.69 ± 3.28), PTC (350 ± 193 vs. 323 ± 139) and FSH (74.04 ± 25.46 vs. 63.56 ± 26.46) values, (*P* < 0.05). All anthropometric measures were significantly different between the two groups (*P* < 0.001). Meanwhile, there were no differences between the groups with and without sarcopenia regarding TSH, PCT, 25(OH)VD, glucose, CHOL, LDL, and AST. We also calculated the A/G and AST/ALT ratios, and both had significant differences (*P* < 0.01).

**Table 1:** Metabolic characteristics and anthropometric measures of study participants

	No sarcopenia (n = 2340)	Sarcopenia (n = 497)	*P* value
AGE (years)	60.4 ± 7.4	67.2 ± 9.0	0.000*
TSH (mIU/L)	3.60 ± 2.91	3.58 ± 5.07	0.928
FT3/(pmol/L)	4.52 ± 1.43	4.30 ± 0.73	0.000*
FT4/(pmol/L)	17.69 ± 3.28	18.06 ± 2.98	0.014*
PTC/(nmol/L)	323 ± 139	350 ± 193	0.000*
FSH/(IU/L)	63.56 ± 26.46	74.04 ± 25.46	0.000*
PCT/(mmol/L)	0.19 ± 0.05	0.18 ± 0.05	0.050
25(OH)VD/(nmol/L)	17.95 ± 5.71	17.44 ± 6.31	0.103
Insulin/(mmol/L)	8.96 ± 7.18	6.21 ± 4.17	0.000*
Glucose/(mmol/L)	5.49 ± 1.57	5.40 ± 1.76	0.328
TP/(g/L)	72.3 ± 6.3	71.6 ± 5.3	0.009*
ALB/(g/L)	44.5 ± 2.9	43.3 ± 3.3	0.000*
PA/(g/L)	278.38 ± 148.23	257.31 ± 126.01	0.001*
A/G	1.63 ± 0.26	1.57 ± 0.24	0.000*
ALT/(U/L)	27.4 ± 19.3	21.6 ± 13.3	0.000*
AST/(U/L)	28.9 ± 13.7	28.7 ± 15.3	0.717
AST/ALT	1.19 ± 0.38	1.46 ± 0.46	0.000*
CHOL/(mmol/L)	4.89 ± 0.90	4.89 ± 1.01	0.962
TG/(mmol/L)	1.92 ± 1.76	1.74 ± 1.65	0.029*
HDL/(mmol/L)	1.29 ± 0.29	1.39 ± 0.35	0.000*
LDL/(mmol/L)	2.72 ± 0.87	2.71 ± 0.86	0.882
VLDL/(mmol/L)	0.87 ± 0.80	0.79 ± 0.75	0.030*
^#^Weight/kg	61.47 ± 9.17	48.53 ± 7.5	0.000*
^#^Height/cm	153.32 ± 5.9	148.11 ± 5.6	0.000*
^#^BMI/(kg/m^2^)	26.2 ± 3.7	22.2 ± 3.6	0.000*
Gait speed/(m/s)	5.12 ± 2.88	6.29 ± 3.08	0.000*
^#^Handgrip/kg	19.14 ± 5.53	14.54 ± 3.81	0.000*
^#^ASM/kg	14.09 ± 4.30	10.64 ± 3.23	0.000*
^#^ASMI/(kg/m^2^)	6.42 ± 0.62	5.21 ± 0.36	0.000*

TSH, thyroid-stimulating hormone; FT3, free triiodothyronine; FT4, free tetraiodothyronine; PTC, adrenal cortisol; FSH, follicle-stimulating hormone; PCT, procalcitonin; TP, total protein; ALB, albumin; PA, prealbumin; A/G, albumin to globulin ratio; ALT, alanine aminotransferase; AST, aspartate aminotransferase; AST/ALT, alanine aminotransferase to aspartate aminotransferase ratio; CHOL, cholesterol; TG, triglyceride; HDL, high-density lipoprotein; LDL, low-density lipoprotein; VLDL, very low density lipoprotein; BMI, body mass index (weight/ height^2^); ASM, appendicular skeletal muscle mass; ASMI, appendicular skeletal muscle mass index (ASM/height^2^). *P < 0.05, ^#^*t*-test was used to compare two groups

### Multivariate regression of biochemical parameters and muscle mass

Based on the findings of univariate analysis, the following parameters were entered in the multivariate logistic regression model: ALB, PA, TP, A/G, insulin, AST/ALT, FSH, FT3, FT4, PTC, TG, HDL, and VLDL. After adjustment for age, the odds ratios (ORs) of low muscle mass showed that AST/ALT ratio (OR = 1.819, [95% CI: 1.417–2.335] *P* < 0.001), FT4 (OR = 1.068, [95% CI: 1.026–1.112] *P* < 0.001), HDL (OR = 1.032, [95% CI: 1.102–1.053] *P* = 0.002), FSH (OR = 1.016, [95% CI: 1.012–1.020] *P* < 0.001), PTC (OR = 1.001, [95% CI: 1.000-1.002] *P* = 0.005), PA (OR = 0.998, [95% CI: 0.996–0.999] *P* = 0.008), insulin (OR = 0.854, [95% CI: 0.827–0.881] *P* < 0.001), and A/G (OR = 0.261, [95% CI: 0.070–0.972] *P* = 0.045) were identified as risk factors for low skeletal muscle **(**Table 2**)**.

**Table 2:** Multivariate regression of biochemical parameters and muscle mass

	Unadjusted OR	95%CI	*P* value	Adjusted OR	95%CI	*P* value
ALB	1.019	0.889–1.169	0.785	1.071	0.949–1.208	0.268
PA	0.997	0.995–0.999	0.001*	0.998	0.996–0.999	0.008*
TP	0.996	0.916–1.082	0.917	0.993	0.924–1.067	0.848
A/G	0.275	0.062–1.217	0.089	0.261	0.070–0.972	0.045*
AST/ALT	2.464	1.945–3.120	0.000*	1.819	1.417–2.335	0.000*
Insulin	0.873	0.846-0.900	0.000*	0.854	0.827–0.881	0.000*
FSH	1.015	1.011–1.019	0.000*	1.016	1.012–1.020	0.000*
FT3	0.79	0.681–0.916	0.002*	0.881	0.770–1.008	0.066
FT4	1.087	1.045–1.131	0.000*	1.068	1.026–1.112	0.001*
PTC	1.001	1.001–1.002	0.000*	1.001	1.000-1.002	0.005*
TG	1.036	0.879–1.220	0.674	0.884	0.869–1.214	0.756
HDL	1.038	1.018–1.059	0.000*	1.032	1.102–1.053	0.002*
VLDL	0.799	0.257–2.480	0.698	0.844	0.264–2.695	0.775

Model was adjusted for age. FT3, free triiodothyronine; FT4, free tetraiodothyronine; PTC, adrenal cortisol; FSH, follicle-stimulating hormone; TP, total protein; ALB, albumin; PA, prealbumin; A/G, albumin to globulin ratio; AST/ALT, alanine aminotransferase to aspartate aminotransferase ratio; TG, triglyceride, HDL, high-density lipoprotein; VLDL, very low-density lipoprotein; *P < 0.05

### The predictive value of parameters for muscle mass

**Fig. 1:**
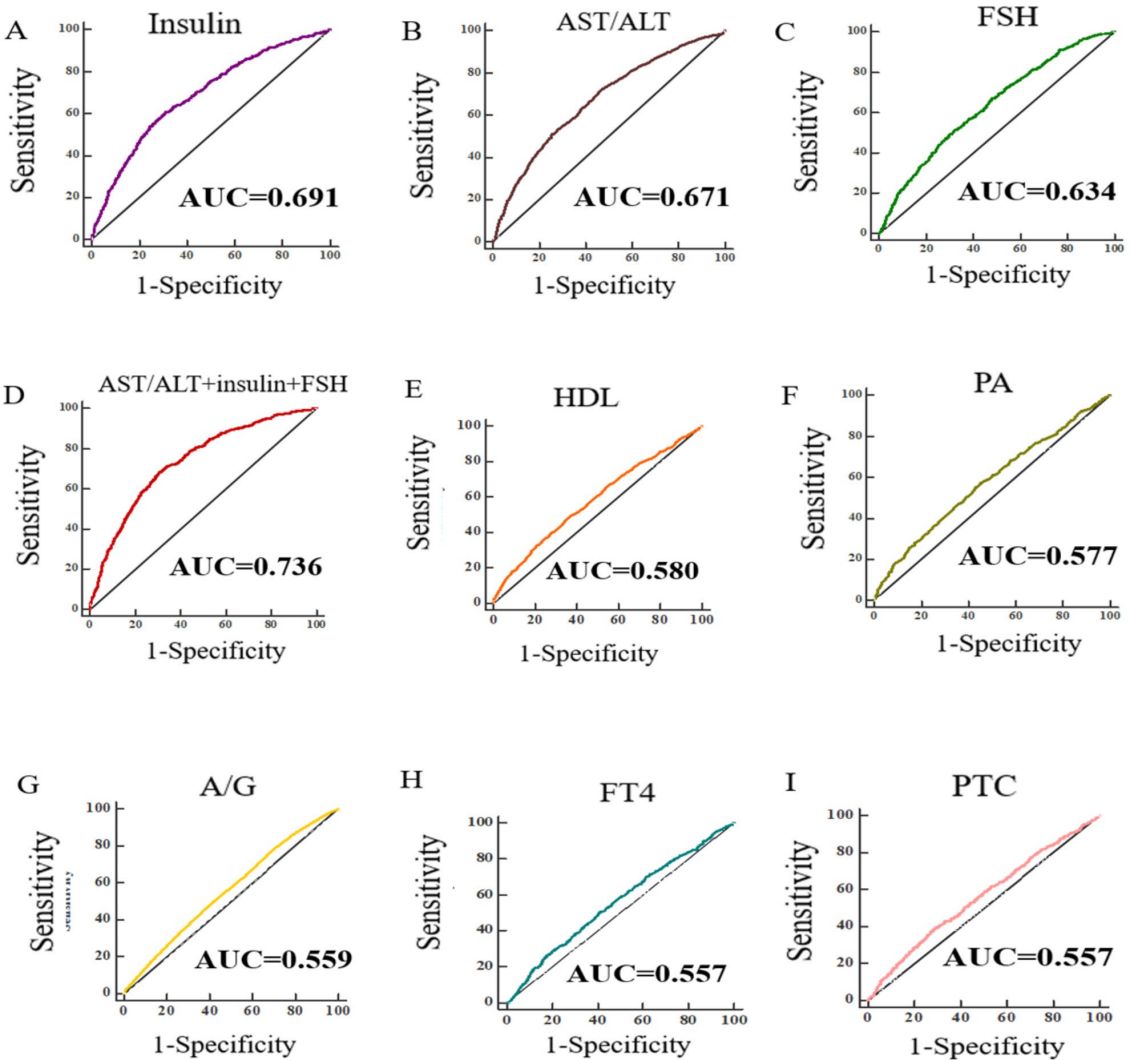
Receiver operating characteristic curves of different biochemical indicators in skeletal muscle mass. AST/ALT, alanine aminotransferase to aspartate aminotransferase ratio; FSH, follicle-stimulating hormone; HDL, high-density lipoprotein; PA, prealbumin; A/G, albumin to globulin ratio; FT4, free tetraiodothyronine; PTC, adrenal cortisol

We established receiver operating characteristic curves (ROC) and assessed the diagnostic performance of the biochemical indicators reflecting the low muscle mass in the female sarcopenia cohort by the area under the curve (AUC). Fasting insulin had the best diagnostic performance AUC = 0.691 [95% CI: 0.674–0.708], followed by AST/ALT ratio AUC = 0.671 [95% CI: 0.653–0.688], FSH AUC = 0.634 [95% CI: 0.616–0.652], HDL AUC = 0.580 [95% CI: 0.561–0.598], PA AUC = 0.577 [95% CI: 0.559–0.595], A/G AUC = 0.559 [95% CI: 0.541–0.577], FT4 AUC = 0.557 [95% CI: 0.538–0.575], and PTC AUC = 0.557 [95% CI: 0.538–0.575]. Then, we combined parameters of the top three AUCs and found that the AUC of the mixture could reach 0.736 [95% CI: 0.720–0.753] **(**Fig. 1**)**.

Meanwhile, we also stratified participants by age to eliminate the confounding factor, and we found that sarcopenia group had higher values of AST/ALT ratio and FSH, but lower insulin and ASMI than no sarcopenia group (*P* < 0.01) **(**Table 3**)**. Then, we stratified participants according to the cutoff values of FSH (72.6), insulin (6.06), and AST/ALT ratio (1.35). As shown in Table 4, nonsarcopenia participants had higher muscle mass (*P* < 0.001) and lower handgrip (*P*=0.655) in the lower-FSH group than in the higher-FSH group. However, in the sarcopenia cohort, there was no difference in handgrip strength (*P* = 0.805) or muscle mass (*P* = 0.308) between the lower and higher FSH groups. In the insulin subgroup, patients without sarcopenia had higher handgrip strength (*P* = 0.013) and muscle mass (*P* < 0.001) in the higher-insulin group than in the lower-insulin group, but in the sarcopenia cohort, no difference was found in handgrip strength (*P* = 0.534), but sarcopenia with high insulin had higher muscle mass than low insulsin (*P*=0.001). Meanwhile, in the AST/ALT subgroups, patients without sarcopenia had higher handgrip strength (*P* < 0.001) and muscle mass (*P* < 0.001) in the lower-AST/ALT group than in the higher-AST/ALT group, but in the sarcopenia cohort, no difference was found in handgrip strength (*P* = 0.348), but sarcopenia with low AST/ALT had higher muscle mass than high AST/ALT (*P*=0.001).

**Table 3:** Biochemical parameters and muscle mass stratified by age

		sarcopenia	No sarcopenia	*P* value
**AGE:50 ~ 59**	Insulin/(mmol/L)	4.90(3.71,7.00)	7.25(5.22,10.4)	0.000*
	AST/ALT	1.19(1.02,1.41)	1.09(0.89,1.31)	0.001*
	FSH/(IU/L)	77.4(57.4,94.6)	63.2(43.6,82.1)	0.000*
	ASMI/(kg/m^2^)	5.40(5.20,5.60)	6.40(6.00.6.90)	0.000*
**AGE:60 ~ 69**	Insulin/(mmol/L)	5.40(3.43,8.03)	8.06(5.63,11.3)	0.000*
	AST/ALT	1.36(1.13,1.64)	1.14(0.95,1.38)	0.000*
	FSH/(IU/L)	68.9(54.2,81.5)	62.0(49.1,77.2)	0.001*
	ASMI/(kg/m^2^)	5.30(5.05,5.50)	6.30(6.00,6.70)	0.000*
**AGE:70 ~ 79**	Insulin/(mmol/L)	5.23(3.68,8.22)	8.37(5.25,11.8)	0.000*
	AST/ALT	1.50(1.27,1.83)	1.27(1.05,1.54)	0.000*
	FSH/(IU/L)	71.8(55.8,95.4)	60.1(50.8,76.1)	0.000*
	ASMI/(kg/m^2^)	5.20(5.00,5.40)	6.20(5.90,6.60)	0.000*
**AGE > 80**	Insulin/(mmol/L)	5.22(3.46,7.17)	9.78(6.25,15.0)	0.000*
	AST/ALT	1.77(1.45,2.14)	1.35(1.11,1.69)	0.003*
	FSH/(IU/L)	73.8(62.9,92.3)	69.0(51.4,95.1)	0.568
	ASMI/(kg/m^2^)	5.00(4.70,5.40)	6.10(5.90,6.40)	0.000*

AST/ALT, alanine aminotransferase to aspartate aminotransferase ratio; FSH, follicle-stimulating hormone; ASMI, appendicular skeletal muscle mass index. *P <0.05

**Table 4:** Muscle mass associated components in the female sarcopenia cohort stratified by the FSH, insulin, and AST/ALT ratio values

		Lower FSH	Higher FSH	*P* value
No sarcopenia		n = 1538	n = 792	
	Handgrip (kg)	19.1 ± 5.5	19.2 ± 5.6	0.655
	Muscle mass (kg/m^2^)	6.52 ± 0.62	6.22 ± 0.55	0.000*
Sarcopenia		n = 250	n = 244	
	Handgrip (kg)	14.6 ± 4.0	14.5 ± 3.7	0.805
	Muscle mass (kg/m^2^)	5.22 ± 0.38	5.20 ± 0.35	0.308
		**Lower insulin**	**Higher insulin**	***P*** **value**
No sarcopenia		n = 757	n = 1583	
	Handgrip (kg)	18.7 ± 5.5	19.3 ± 5.5	0.013*
	Muscle mass (kg/m^2^)	6.23 ± 0.58	6.51 ± 0.61	0.000*
Sarcopenia		n = 303	n = 193	
	Handgrip (kg)	14.5 ± 3.8	14.7 ± 3.9	0.534
	Muscle mass (kg/m^2^)	5.16 ± 0.39	5.28 ± 0.30	0.001*
		**Lower AST/ALT**	**Higher AST/ALT**	***P*** **value**
No sarcopenia		n = 1733	n = 607	
	Handgrip (kg)	19.4 ± 5.5	18.4 ± 5.5	0.000*
	Muscle mass (kg/m^2^)	6.49 ± 0.64	6.23 ± 0.51	0.000*
Sarcopenia		n = 217	n = 279	
	Handgrip (kg)	14.7 ± 3.7	14.4 ± 3.9	0.348
	Muscle mass (kg/m^2^)	5.28 ± 0.31	5.16 ± 0.39	0.001*

AST/ALT, alanine aminotransferase to aspartate aminotransferase ratio; FSH, follicle-stimulating hormone

The cutoff values of FSH, insulin, and AST/ALT ratio are 72.6 IU/L, 6.06 mmol/L, and 1.35, respectively. *P <0.05

## Discussion

In this study, we first identified some biochemical markers related to sarcopenia among elderly Chinese participants from the WCHAT study and found that participants with sarcopenia had significantly lower FT3, insulin, TP, ALB, PA, A/G ratio, ALT, TG, and VLDL concentrations (*P* = 0.009 for TP; *P* = 0.029 for TG; *P* = 0.030 for VLDL; *P* < 0.001 for the other biomarkers). Compared with those without sarcopenia, those with sarcopenia had significantly higher FT4, PTC, FSH, AST/ALT ratio, and HDL concentration (*P* = 0.014 for FT4, *P* < 0.001 for the other biomarkers). Our results showed that sarcopenia participants had malnutrition. Previous studies also revealed that supplementation with essential amino acids can prevent sarcopenia in older individuals [22, 23]. In addition, thyroid hormones also play a role in skeletal muscle contractile function and muscle regeneration [24]. A reduction in 5`-deiodinas with aging results in a higher FT4 but FT3 level in sarcopenia patients [25]. An increase in adrenal cortisol in sarcopenia may stimulate muscle catabolism by inducing atrogin-1 and MuRF-1 in the ubiquitin-proteasome system, thus causing protein degradation of skeletal muscle [26]. In terms of lipid metabolism, Jaekyung et al. confirmed that TG and HDL levels had a positive and negative correlation with sarcopenia, which is consistent with our results [27].

Multivariate regression of biochemical parameters and muscle mass showed that fasting insulin (OR = 0.854), FSH (OR = 1.016), and AST/ALT ratio (OR = 1.819) were independent risk factors for low muscle mass in female sarcopenia, but the associations were weak. Meanwhile. Based on the findings of univariate and multivariate analysis, we also established ROCs to identify biochemical markers that reflect skeletal muscle mass in the female sarcopenia cohort and found that the AUC of insulin was the highest, followed by the AST/ALT ratio and FSH (0.691, 0.671, and 0.634, respectively), and the AUC of the mixture of the above three reached 0.736. Meanwhile, we divided participants into lower and higher AST/ALT ratio (1.35), insulin (6.06 mmol/L), and FSH (72.6 IU/L) groups according to their cutoff values. The higher FSH levels group had low muscle mass than that of the higher FSH levels group among nonsarcopenia participants, however, no difference had been found in muscle mass between the higher and the lower FSH levels group among sarcopenia participants (*P* = 0.308). Meanwhile, we also stratified participants by age and found that there was no difference of FHS levels between groups at the age of more than 80 years old (*P* = 0.568). These results showed that FSH was a good indicator for appendicular lean mass in women, and the effects of FSH were prominent. However, other studies demonstrated that strong associations were observed in the men, whereas in the women there was no association between FSH and muscle mass [15, 28]. Thus, further studies for FSH on muscle mass, muscle strength and function should be explored in the future day. Skeletal mass accounts for 40–50% of lean body mass in adult humans, and therefore for the majority of whole-body insulin-stimulated glucose disposal. In our study, patients with sarcopenia had higher insulin levels. Meanwhile, insulin levels were associated with muscle mass among sarcopenia and nonsarcopenia group. Sugimoto et al. have also revealed that the use of insulin was significantly associated with the increase in muscle mass and gait speed in type 2 diabetes [29]. All results elucidated that there was a strong relationship between insulin and muscle mass. In our previous study, we verified AST/ALT had a good diagnostic performance for sarcopenia [30, 31]. In this study, we found that AST/ALT ratio was associated with skeletal muscle mass among sarcopenia and nonsarcopenia groups, which is consistent with the results by Lasman et al. [32]. To sum up, the current research is a cross-sectional study using baseline data of the WCHAT study and data were collected from four provinces in West China, including Yunnan, Guizhou, Sichuan, and Xinjiang, therefore, our results may be generalizable. However, we should also acknowledge the limitation of the present study. In this study, although the relationship between biomarkers and muscle mass in women sarcopenia cohort was observed, as a cross-sectional study, the causality of the relationship cannot be established, thus, further studies are needed.

## Conclusion

In this cross-sectional study of elderly Chinese females aged over 50 years from the WCHAT, FSH, insulin, and the AST/ALT ratio were associated with sarcopenia and risk factors for low muscle mass.

## Data Availability

The data-set generated and analyzed during the current study will be available from the corresponding author on a reasonable request.
